# Influence of Obesity Class on Clinical Outcomes in Alcoholic Hepatitis: A National Cohort Study of Mortality, Complications, and Resource Use

**DOI:** 10.1002/jgh3.70166

**Published:** 2025-04-15

**Authors:** Ali Jaan, Mostafa Suhail Najim, Umer Farooq, Ashish Dhawan, Hassan Nawaz, Vinay Jahagirdar, Hassam Ali, Sushil Ahlawat

**Affiliations:** ^1^ Division of Internal Medicine Rochester General Hospital Rochester USA; ^2^ Division of Internal Medicine Unity Hospital Rochester USA; ^3^ Division of Gastroenterology Saint Louis University Missouri USA; ^4^ Division of Internal Medicine Gian Sagar Medical College and Hospital Patiala district India; ^5^ Division of Internal Medicine Nishtar Medical University Multan Pakistan; ^6^ Division of Gastroenterology Virginia Commonwealth University Richmond USA; ^7^ Division of Gastroenterology East Carolina University/ECU Health Medical Center Greenville USA; ^8^ Division of Gastroenterology SUNY Downstate Health Sciences University Brooklyn USA

**Keywords:** alcoholic hepatitis, Hepatorenal syndrome, National Readmission Database, obesity

## Abstract

**Background & Aims:**

Alcoholic hepatitis (AH) is a severe manifestation of alcoholic liver disease with high morbidity and mortality. This study used the 2016–2020 National Readmission Database to investigate how obesity influences AH outcomes.

**Methods:**

Adult hospitalizations were categorized as those without obesity, Class 1 obesity (BMI 30–34.9), Class 2 obesity (BMI 35–39.9), or Class 3 obesity (BMI ≥ 40). We compared mortality, complications, and resource utilization across these groups using regression models.

**Results:**

Among 82 367 AH admissions, 4.09% had Class 1 obesity, 2.73% had Class 2 obesity, and 4.02% had Class 3 obesity. After adjusting for confounders, Class 3 obesity was associated with higher odds of mortality (Odds ratio OR = 1.74; 95% CI: 1.40–2.17; *p* < 0.01), septic shock (OR = 2.27; 95% CI: 1.60–3.22; *p* < 0.01), hepatic encephalopathy (OR = 2.53; 95% CI: 1.15–5.56; *p* = 0.02), and intensive care unit (ICU) admission (OR = 1.93; 95% CI: 1.57–2.36; *p* < 0.01). All obesity classes had increased associations with hepatorenal syndrome. No significant differences emerged for spontaneous bacterial peritonitis or variceal bleeding. Resource utilization rose with increasing obesity severity, with Class 3 obesity having a 1.84‐day longer adjusted length of stay (*p* < 0.01) and an additional $20 174 in total hospitalization charges (*p* < 0.01) compared with hospitalizations without obesity.

**Conclusions:**

Class 3 obesity conferred the greatest burden of mortality, complications, and healthcare costs among hospitalizations with AH. Further research is warranted to clarify the intricate interplay between obesity and AH.

**Abbreviations**
AH: Alcoholic hepatitis
aHR: Adjusted hazard ratio
ALD: Alcoholic liver disease
aOR: Adjusted odds ratio
BMI: Body mass index
CCI: Charlson comorbidity index
CI: Confidence interval
HE: Hepatic encephalopathy
HRS: Hepatorenal syndrome
ICD: International classification of diseases
ICU: Intensive care unit
LOS: Length of stay
MDF: Maddrey's discriminant function
MELD‐Na: Model for end‐stage liver disease‐Sodium
NHANES: National health and nutrition examination survey
NRD: National Readmission Database
SBP: Spontaneous bacterial peritonitis
THC: Total hospitalization cost
VUGIB: Variceal upper gastrointestinal bleeding

## 1 Introduction

Alcohol‐associated liver disease (ALD) encompasses various stages of liver injury related to excessive alcohol intake, starting with alcoholic steatosis and progressing to alcoholic hepatitis (AH), eventually leading to irreversible alcoholic cirrhosis [1]. AH involves an inflammatory process that can manifest as acute liver failure and has a poor prognosis. “Non‐severe” forms of AH carry a 28‐day mortality of 6%, whereas over 50% of patients with “severe” AH die within 6 months [2, 3]. Despite the current advancements in the medical field, the mortality burden of this disease has not improved over time [4].

On the other hand, the prevalence of obesity is on the rise globally, and around 38% of the United States population is estimated to have obesity [5]. Obesity has detrimental effects on health and is associated with significantly increased all‐cause mortality, regardless of grade, when compared to individuals with a normal body mass index (BMI) [6]. A population‐based study involving the Third National Health and Nutrition Examination Survey (NHANES III) participants and linked mortality data found obesity to be an independent predictor of liver‐related mortality in patients with chronic liver disease [7]. Moreover, there is growing evidence that obesity and alcohol have a synergistic detrimental effect on hepatic cells, resulting in an elevated risk of hepatocellular carcinoma [8, 9].

In light of the increasing prevalence of obesity and the paucity of clinical data on its influence on AH outcomes, it is crucial to comprehend this relationship. This study aimed to investigate the prevalence and clinical implications of obesity on AH outcomes in the United States using the National Readmission Database (NRD).

## 2 Methods and Materials

We performed a retrospective cohort study utilizing data for the years 2016–2020 from the NRD [10]. The NRD is the largest publicly available all‐payer inpatient healthcare database, covering 31 states and more than 60% of the U.S. population [10]. This data contains information on all hospital stays, regardless of the expected payer for the hospital stay. It is sampled from the State Inpatient Database and contains data on approximately 17 million hospitalizations annually. A 20% stratified sample was gathered from all U.S. community hospitals, excluding rehabilitation and long‐term care hospitals. Each discharge from the resultant data was then weighted (weight equals the total number of discharges from all acute care hospitals in the United States divided by the number of discharges included in the 20% sample) to make the NRD nationally representative. When weighted, the NRD data estimate approximately 32 million hospitalizations across the U.S. The NRD aims to make regional and national estimates of healthcare utilization, cost, quality, and outcomes in the USA. It contains more than 100 de‐identified clinical and non‐clinical elements for each hospital stay at the hospital and patient level.

### 2.1 Study Population

We included adult hospitalizations (aged ≥ 18 years) with a principal diagnosis of AH, utilizing the International Classification of Diseases, Tenth Revision, and Clinical Modifications (ICD‐10‐CM) codes (Table S1). The hospitalizations were further classified into four groups based on the presence and severity of obesity, that is, non‐obese (BMI < 30), Class 1 obesity (BMI 30–34.9), Class 2 obesity (BMI 35–39.9), and Class 3 obesity (BMI ≥ 40) [11]. Hospitalizations without obesity were used as references for the statistical analysis. To accurately identify all patients with AH and not miss any diagnosis code, we reviewed previously published literature on NRD for AH [12]. The STROBE checklist and ICD‐10‐CM diagnosis and procedure codes used in this study are listed in the Supporting Information. Although our study utilized the NRD database, which contains de‐identified data, approval was nevertheless sought from the institutional review board. Given the nature of the data, this study was deemed exempt from full review.

### 2.2 Study Variables and Outcomes

We aimed to assess the impact of obesity on AH outcomes. The primary outcome was all‐cause mortality. Secondary outcomes evaluated included septic shock, vasopressor requirement, need for mechanical ventilation, and admission to the intensive care unit (ICU). AH‐specific complications, including spontaneous bacterial peritonitis (SBP), variceal upper gastrointestinal bleeding (VUGIB), hepatic encephalopathy (HE), and hepatorenal syndrome (HRS), were also assessed. Furthermore, we evaluated resource utilization using measures such as total hospitalization charges (THC), length of stay (LOS), and discharge disposition (home vs. rehabilitation centers) among the groups.

### 2.3 Statistical Analysis

STATA, version 14.2 (StataCorp., College Station, Texas, USA) was used for the analyses. Multivariate analyses were performed using confounders with a *p* value ≤ 0.2 on univariate regression analysis [13]. In addition, we incorporated variables that were considered clinically significant for the outcome, regardless of their statistical significance in the univariate analysis. The variables included in the final regression analysis for CDI were age, gender, Charlson comorbidity index (CCI), insurance status (Medicare, Medicaid, private, and uninsured), hospital bed size (small, medium, and large), hospital location (rural vs. rural), hospital teaching status, and end‐stage renal disease (ESRD). Prior to applying descriptive statistics, the normality of individual variables was assessed through the Kolmogorov–Smirnov test. The continuous variables, specifically LOS and THC, followed a Poisson distribution. Consequently, we employed the Mann–Whitney U test to assess means and Poisson regression to assess the adjusted mean difference in the linear regression model. To analyze binary outcomes, logistic regression was utilized. All *p* values were two‐sided, and the threshold for statistical significance was set at *p* < 0.05.

## 3 Results

### 3.1 Patient Characteristics

**FIGURE 1:**
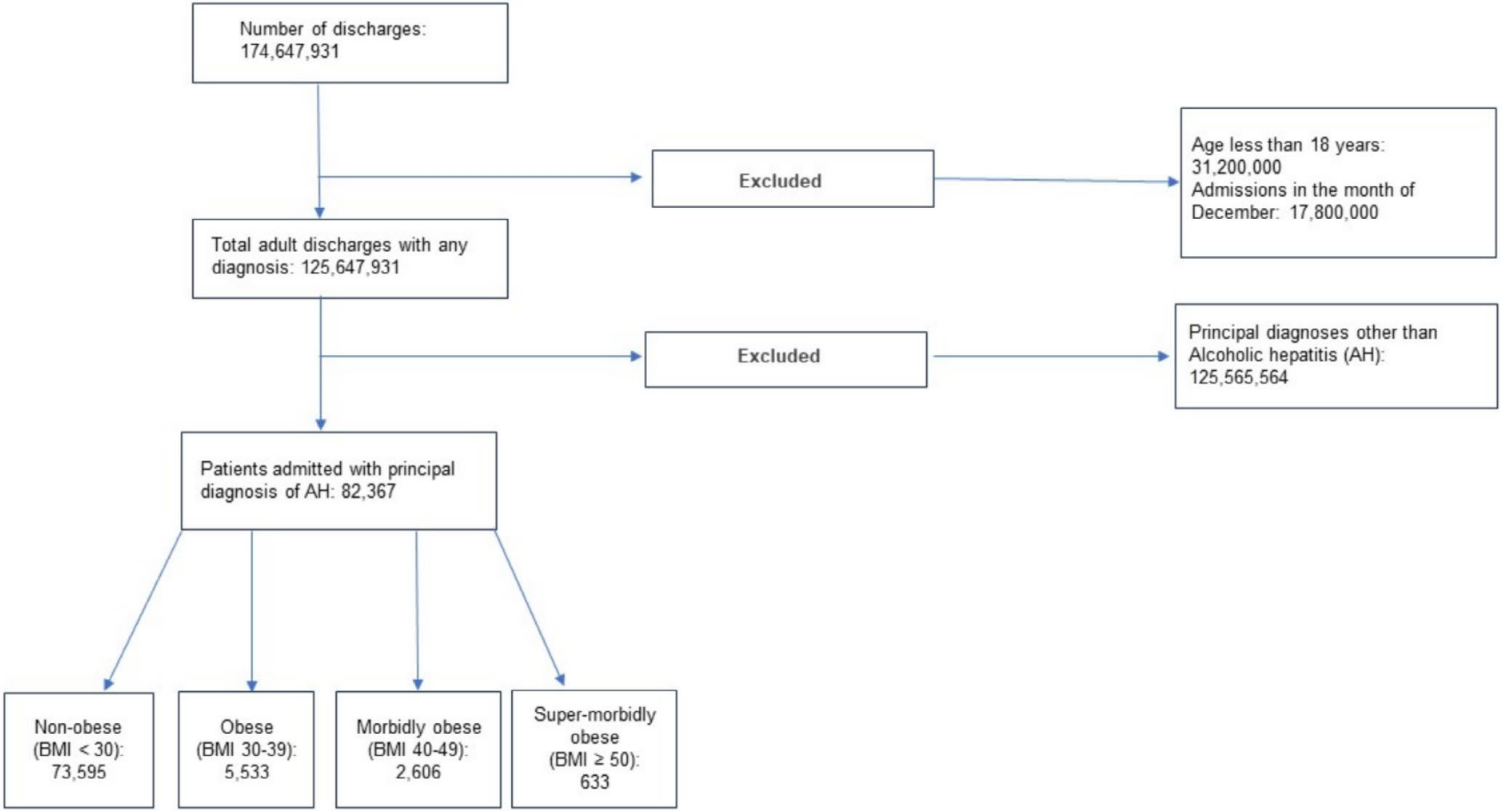
Study participants flowchart.

We analyzed 82 367 adult hospitalizations admitted with AH. Among these, 4755 (4.09%) had Class 1 obesity, 3201 (2.73%) had Class 2 obesity, and 4651 (4.02%) had Class 3 obesity (Figure 1). Hospitalizations with higher obesity classes were more likely to have a CCI of ≥ 3, with prevalence increasing from 4.60% in the non‐obese group to 7.73% in Class 3 obesity (*p* < 0.01) (Table 1). Similarly, hospitalizations in higher obesity classes were less frequently covered by Medicaid insurance, with coverage rates decreasing from 39.33% in non‐obese patients to 36.59% and 34.83% in Class 2 and Class 3 obesity groups, respectively (*p* < 0.01). The prevalence of congestive heart failure rose from 13.13% in hospitalizations without obesity to 15.13% and 21.52% in Class 2 and Class 3 obesity groups, respectively (*p* < 0.01) (Table 2).

**TABLE 1:** Demographics of alcoholic hepatitis hospitalizations, stratified by severity of obesity.

Alcoholic hepatitis +Age > =18: (*n*=82,367)					
Baseline characteristics	Non‐obese	Obesity Class 1 (BMI 30–34.9)	Obesity Class 2 (BMI 35–39.9)	Obesity Class 3 (BMI ≥ 40)	*p*
Number of patients					
Age [mean (95% confidence interval)] (years)					
Female gender, %	40.08	35.56	38.59	41.25	0.15
Charlson comorbidity index, %					< 0.01
1	78.34	76.74	71.90	69.69	
2	17.06	15.78	21.50	22.58	
≥ 3	4.60	7.48	6.60	7.73	
Median household income in the patient's zip code (quartile), %					0.06
1st (0–25th)	30.08	26.09	29.94	24.07	
2nd (26th–50th)	27.38	28.25	29.42	29.37	
3rd (51st–75th)	24.56	26.76	21.61	27.15	
4th (76th–100th)	17.98	18.91	19.03	19.41	
Insurance status, %					< 0.01
Medicare	21.99	22.00	21.47	17.24	
Medicaid	39.33	32.43	34.83	36.59	
Private	27.68	34.25	35.53	39.21	
Uninsured	11.00	11.32	8.18	6.96	
Hospital bed size, %					0.96
Small	17.22	16.98	16.83	17.38	
Medium	27.77	26.94	28.57	25.78	
Large	55.01	56.08	54.60	56.84	
Hospital teaching status, %					0.57
Non‐teaching	24.55	22.94	26.69	24.13	
Teaching	75.45	77.06	73.31	75.87	
Hospital location, %					0.54
Rural	4.94	4.19	5.78	5.62	
Urban	95.06	95.81	94.22	94.38	

**TABLE 2:** Comorbid status of alcoholic hepatitis hospitalizations, stratified by severity of obesity.

Alcoholic hepatitis + Age > =18: (*n*=82,367)					
Comorbidities	Non‐obese	Obesity Class 1 (BMI 30–34.9)	Obesity Class 2 (BMI 35–39.9)	Obesity Class 3 (BMI ≥ 40)	*p*
Congestive heart failure, %	13.13	11.30	15.13	21.52	< 0.01
End‐stage renal disease, %	1.55	1.54	1.16	3.00	0.09
History of myocardial infarction, %	5.16	6.60	4.92	4.57	0.47
History of cerebrovascular disease, %	2.40	1.35	1.12	1.86	0.15
Peripheral vascular disease, %	4.78	4.45	5.13	4.15	0.86
Chronic obstructive pulmonary disease, %	41.81	36.34	39.08	37.69	0.01
Uncomplicated diabetes, %	23.65	29.12	34.92	31.98	< 0.01
Complicated diabetes, %	5.81	10.28	12.19	10.18	< 0.01
Rheumatoid arthritis, %	3.20	1.82	0.63	2.99	< 0.01
Peptic ulcer disease, %	8.81	9.39	6.14	9.26	0.29
Dementia, %	1.13	1.46	0.43	0.15	0.06
Metastatic disease, %	0.81	0.40	0.12	0.22	0.02
AIDS, %	0.77	0.13	0.28	0.07	0.02
Hemiplegia/Paraplegia, %	0.44	0.60	0.17	0.83	0.39
Chronic kidney disease, %	12.41	15.91	13.21	13.14	0.09

Abbreviation: AIDS, Acquired immunodeficiency syndrome.

### 3.2 Mortality and Morbidity

**FIGURE 2:**
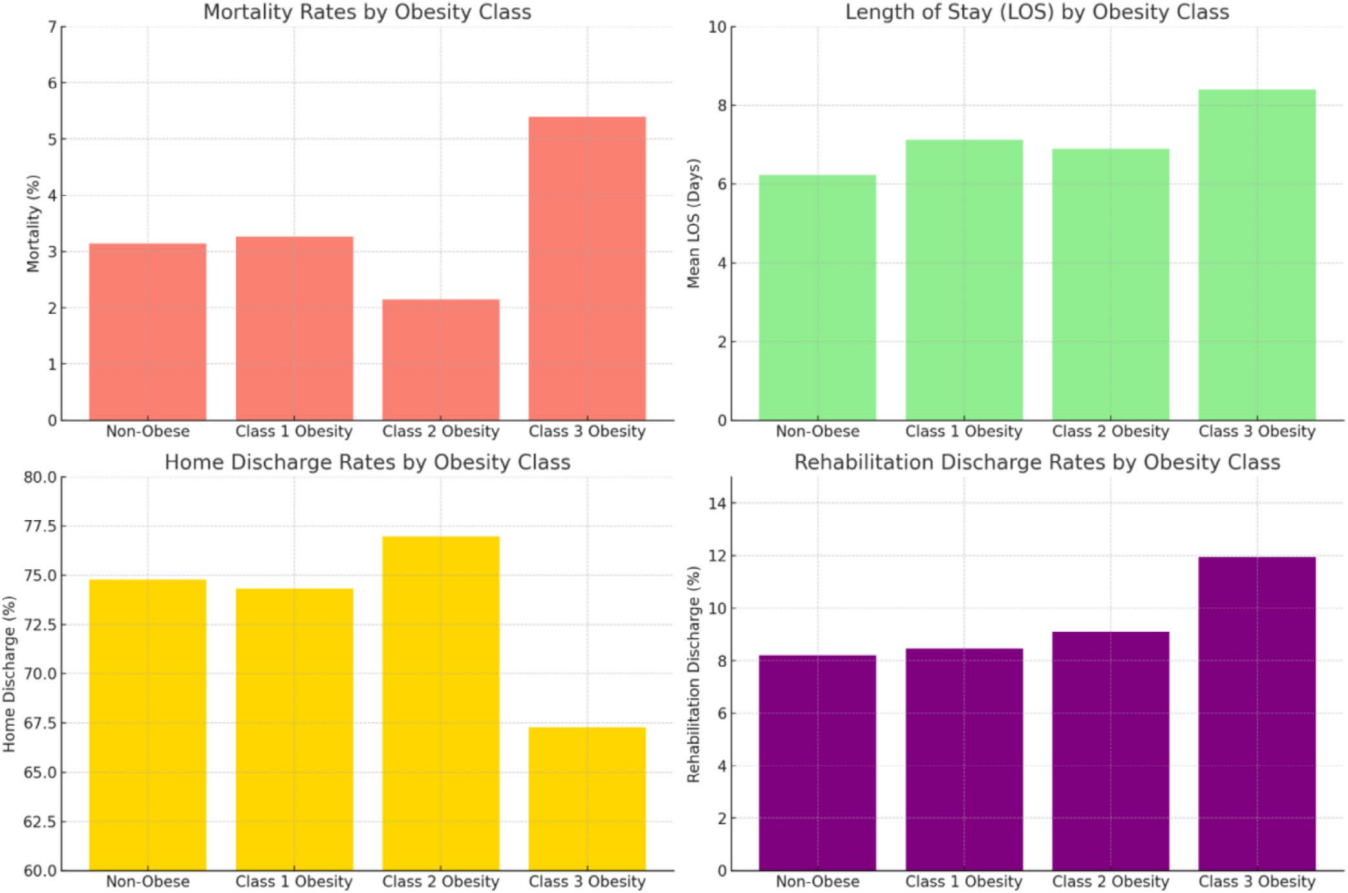
Clinical outcomes and discharge patterns by obesity class in patients with alcoholic hepatitis.

Among hospitalized patients with AH, those with Class 3 obesity (BMI ≥ 40) demonstrated the highest unadjusted in‐hospital mortality rate (5.40%) compared to non‐obese individuals (3.15%), as well as those with Class 1 (3.26%) and Class 2 obesity (2.15%) (Figure 2). After adjusting for confounders, Class 3 obesity was significantly associated with greater odds of in‐hospital mortality (adjusted odds ratio OR = 1.74; 95% CI: 1.40–2.17; *p* < 0.01). Class 3 obesity also showed elevated risks of septic shock (OR = 2.27; 95% CI: 1.60–3.22; *p* < 0.01), mechanical ventilation (OR = 2.02; 95% CI: 1.63–2.49; *p* < 0.01), ICU admission (OR = 1.93; 95% CI: 1.57–2.36; *p* < 0.01), and vasopressor use (OR = 1.73; 95% CI: 1.09–2.73; *p* = 0.02). Notably, HRS was more frequent across all obesity classes, with ORs of 1.31 (Class 1), 1.29 (Class 2), and 2.37 (Class 3). HE was significantly elevated only in Class 3 obesity (OR = 2.53; 95% CI: 1.15–5.56; *p* = 0.02). By contrast, neither SBP nor VUGIB differed significantly across obesity groups (Table 3).

**TABLE 3:** Unadjusted and adjusted hospitalization outcomes of alcoholic hepatitis hospitalizations, stratified by severity of obesity.

Alcoholic hepatitis + age > =18: (*n*=82,367)							
Outcomes	Non‐obese	Obesity Class 1 (BMI 30–34.9)	Obesity Class 2 (BMI 35–39.9)	Obesity Class 3 (BMI ≥ 40)	Obesity Class 1 (BMI 30–34.9)	Obesity Class 2 (BMI 35–39.9)	Obesity Class 3 (BMI ≥ 40)
					Adjusted OR (95% CI, *p*)	Adjusted OR (95% CI, *p*)	Adjusted OR (95% CI, *p*)
Mortality, %	3.15	3.26	2.15	5.40	1.01 (0.72–1.43, 0.94)	0.72 (0.48–1.04, 0.08)	1.74 (1.40–2.17, < 0.01)
Septic shock, %	1.30	1.09	1.00	3.22	0.83 (0.49–1.41, 0.49)	0.77 (0.42–1.40, 0.39)	2.27 (1.60–3.22, < 0.01)
Hepatorenal syndrome, %	4.86	6.73	6.28	11.80	1.31 (1.02–1.67, 0.03)	1.29 (1.01–1.66, 0.04)	2.37 (2.01–2.81, < 0.01)
Vasopressor use, %	0.84	0.82	0.97	1.83	0.95 (0.54–1.67, 0.86)	1.14 (0.63–2.11, 0.65)	1.73 (1.09–2.73, 0.02)
Mechanical ventilation, %	3.05	3.12	2.72	6.71	0.96 (0.68–1.36, 0.82)	0.88 (0.61–1.28, 0.50)	2.02 (1.63–2.49, < 0.01)
Requiring ICU admission, %	3.36	3.39	3.14	7.18	0.95 (0.68–1.32, 0.76)	0.92 (0.66–1.29, 0.64)	1.93 (1.57–2.36, < 0.01)
Spontaneous bacterial peritonitis, %	1.96	1.89	1.35	2.79	0.93 (0.66–1.33, 0.71)	0.68 (0.38–1.20, 0.18)	1.39 (0.98–1.97, 0.06)
Variceal upper GI bleeding, %	1.90	1.75	1.64	1.68	0.84 (0.55–1.27, 0.40)	0.85 (0.49–1.48, 0.57)	0.87 (0.58–1.30, 0.51)
Hepatic encephalopathy, %	0.17	0.04	0.40	0.41	0.21 (0.03–1.55, 0.13)	2.42 (0.79–7.44, 0.12)	2.53 (1.15–5.56, 0.02)

Abbreviations: CI, confidence interval; GI, gastrointestinal; ICU, intensive care unit; R, odds ratio.

### 3.3 Resource Utilization

Regarding resource utilization, the mean LOS rose from 6.23 days in non‐obese to 8.39 days in Class 3 obesity. Adjusted analyses confirmed that Class 3 obesity was associated with a mean LOS increase of 1.84 days (*p* < 0.01). THC also climbed markedly in Class 3 obesity, with an adjusted increase of $20 174 (*p* < 0.01) compared to hospitalizations without obesity. In terms of discharge disposition, Class 3 obesity was tied to a reduced likelihood of home discharge (aOR = 0.68; 95% CI: 0.61–0.76; *p* < 0.01) and a higher probability of discharge to rehabilitation facilities (aOR = 1.65; 95% CI: 1.40–1.95; *p* < 0.01) (Table 4).

**TABLE 4:** Resource utilization of alcoholic hepatitis hospitalizations, stratified by severity of obesity.

Resource utilization	Non‐obese	Obesity Class 1 (BMI 30–34.9)	Obesity Class 2 (BMI 35–39.9)	Obesity Class 3 (BMI ≥ 40)	Obesity Class 1 (BMI 30–34.9)	Obesity Class 2 (BMI 35–39.9)	Obesity Class 3 (BMI ≥ 40)
					Adjusted OR (95% CI, *p*)	Adjusted OR (95% CI, *p*)	Adjusted OR (95% CI, *p*)
Mean LOS, d	6.23	7.12	6.89	8.39	0.74* (0.30–1.17, < 0.01)	0.57* (−0.15–1.29, 0.13)	1.84* (1.32–2.36, < 0.01)
Mean THC, USD	57 020	61 958	60 613	82 250	2309* (−2372–6990, 0.33)	2042* (−4002–8086,0.51)	20174* (11933–28 416, < 0.01)
Home discharge, %	74.76	74.32	76.97	67.27	1.05 (0.93–1.18, 0.42)	1.15 (1.00–1.33, 0.04)	0.68 (0.61–0.76, < 0.01)
Rehabilitation discharge, %	8.20	8.44	9.09	11.95	0.97 (0.80–1.18, 0.78)	1.12 (0.89–1.41, 0.34)	1.65 (1.40–1.95, < 0.01)

Abbreviations: LOS, length of hospital stay; R, odds ratio; THC, total hospitalization charges adjusted; USD, United States dollar.

* Adjusted mean difference (95% CI).

## 4 Discussion

We found that AH hospitalizations with Class 3 obesity had worse outcomes than hospitalizations with BMI < 30, as evidenced by the elevated mortality odds of 1.74. Furthermore, complications such as septic shock, HE, ICU admission, and resource utilization were higher primarily in patients with Class 3 obesity. HRS was found to have higher odds across all levels of obesity.

The two most common processes leading to hepatic steatosis include ALD and metabolic dysfunction‐associated steatotic liver disease (MASLD) [14]. Alcohol consumption in excess (> 7 times per week) can lead to obesity due to its high caloric content and inhibition of fat oxidation, making obesity a common comorbidity in ALD patients [15]. Studies have indicated an overall obesity prevalence ranging from 44.5% to 66% in ALD patients [7, 16]. Stepanova et al., analyzing the NHANES III cohort, found that obesity was associated with increased liver‐related mortality (adjusted hazard ratio [aHR] 16.22 [CI 1.91–137.68]) [7]. Another study evaluating liver disease mortality among two cohorts in Scotland found that obesity acts in synergy with alcohol consumption, leading to increased mortality with an estimated relative excess risk of 5.58 (95% CI, 1.09–10.1) [17]. Parker et al. studied a cohort of 233 AH patients in the United Kingdom and United States and found that obesity increases the short‐term mortality rate of AH by more than two‐fold [18]. This increased mortality risk may be driven by obesity‐related metabolic dysfunction, which exacerbates hepatic inflammation and fibrosis through mechanisms such as insulin resistance and adipokine dysregulation. These large‐scale data are in line with our findings that point toward the increased mortality in AH patients with obesity.

Among the various liver‐specific complications, we found that HRS was significantly increased in patients with BMI ≥ 30. The 2.5‐fold elevated rates of HRS could be the potential driver for the heightened mortality observed in our sample of AH patients with Class 3 obesity. The role of HRS as an independent factor to predict mortality is supported by a retrospective study conducted in the United Kingdom that reported a hazard ratio of 3.842 (95% CI 2.018–7.312, *p* < 0.0001) with a median survival of 0.52 months (95% CI 0.43–0.61) [19]. Asotibe et al., analyzing the National Inpatient Sample (NIS) concluded that obesity increases the odds of HRS in AH patients; however, their results showed that obesity might lower the odds of VUGIB in AH patients [20]. A study that followed a cohort of patients with liver cirrhosis found that patients who were overweight or had obesity developed clinical decompensation at a higher rate than patients with normal BMI. The most common complications were ascites, HE, and variceal bleeding, respectively [21]. In our population of AH patients, we noticed increased odds of HE in AH patients with Class 3 obesity but not in other subgroups. However, we did not find a statistically significant increase in the rates of SBP or VUGIB among the different groups.

The incidence of AH is increasing in the United States, especially among women and lower socioeconomic status individuals [22]. This, in turn, has reflected on the increased impact of AH on the healthcare system over time. The percentage of AH‐related hospitalizations increased from 0.7% to 0.9% between 2011 and 2017 [23]. Most of these patients required tertiary care center admissions in urban areas with THC ranging between $25 242 and $34 874 [23]. This increased burden of AH is paralleled by the uptrending prevalence of obesity in the United States [24]. In our study, we found that obesity is adding to the burden of AH on the healthcare system. AH hospitalizations with Class 3 obesity had higher THC, longer hospital stays, and higher odds of requiring rehabilitation facilities on discharge, thus underscoring the substantial strain on healthcare resources. To our knowledge, this is the first study to investigate the outcomes of obesity in AH hospitalizations using a large nationally representative sample size, outlining the increase in resource utilization with higher BMI. This data emphasizes the need for a comprehensive reevaluation of our current strategies for managing obesity and underscores the importance of intensified efforts in obesity prevention and treatment.

There are a few limitations to our study. Due to the unavailability of laboratory values in the NRD database, we were unable to assess baseline liver disease severity using a model for end‐stage liver disease‐Sodium (MELD‐Na) or Maddrey's Discriminant Function (MDF) scores. Second, the retrospective nature of the study restricted the complete randomization of the cohorts. Despite adjusting for potential confounders using multivariate regression analysis, there is still the possibility of residual confounding. Moreover, we used the NRD, a claims‐based database that has the inherent limitations of incomplete or missing data [25]. Reliance on diagnosis codes instead of clinical parameters can lead to misclassification of diagnoses. Another limitation is the absence of correction in weight for ascites. Corrected BMI (cBMI) after the ascitic fluid was drained is not available in the current database, and BMI may not reflect adiposity in patients with ascites. Understanding the proportion of patients with ascites in this cohort could provide an important context for interpreting BMI‐related findings and its impact on outcomes. Nevertheless, we used ICD‐10 codes for data retrieval, which are more specific than ICD‐9 codes [26]. Finally, given the limitations of the NRD database, we were unable to account for any racial differences.

Our study has several strengths. To our knowledge, this is the first study that comprehensively analyzes the impact of obesity on AH outcomes at the national level in the United States. We employed the NRD database, which incorporates data from a wide range of hospitals across 32 states in the United States. This confers enhanced external validity and generalizability to our study and helps mitigate biases associated with practice patterns observed in single‐ or multicenter studies [27]. Additionally, we accounted for different socioeconomic and hospital factors, including household income estimates and hospitalization costs, which are not feasible in institution‐based studies.

## 5 Conclusion

Our study sheds light on the prevalence of obesity among patients with AH and its effects on clinical outcomes. The findings indicate the increased susceptibility of individuals with AH and Class 3 obesity to heightened mortality risk and complications, such as septic shock, HE, and ICU admission. The likelihood of HRS escalates in tandem with the severity of obesity, while the utilization of hospital resources is predominantly higher in patients with Class 3 obesity. These findings underscore the importance of addressing and managing obesity in patients with AH, not only for its potential contribution to mortality but also due to its association with a spectrum of complications and increased healthcare resource utilization. Further research is crucial for a comprehensive understanding of the intricate impact of obesity on AH severity and outcomes. This knowledge will inform better clinical management and resource allocation across diverse healthcare settings.

## Ethics Statement

Our study utilized the National Readmission Database, which contains de‐identified data; approval was nevertheless sought from the institutional review board. Given the nature of the data, this study was deemed exempt from full review.

## Consent

Not required due to retrospective de‐identified data analysis.

## Conflicts of Interest

The authors declare no conflicts of interest.

## Supporting information


**Table S1.** International classification of diseases, tenth revision (icd‐10) codes used for the study.

## Data Availability

The dataset used for analysis is publicly available at https://hcup‐us.ahrq.gov/nrdoverview.jsp. Analytical methods used are mentioned in the methodology section, and the codes used for this purpose are outlined in the Supporting Information.
