# Supplementary material for: Influence of Obesity Class on Clinical Outcomes in Alcoholic Hepatitis: A National Cohort Study of Mortality, Complications, and Resource Use
Source: JGH Open. 2025 Apr 15;9(4):e70166. doi: 10.1002/jgh3.70166 (PMC11998181; doi:10.1002/jgh3.70166)
Supplement: Supplementary file 1 — Table S1. International classification of diseases, tenth revision (icd‐10) codes used for the study. [file JGH3-9-e70166-s001.docx]

**Supplementary Table 1: International Classification of Diseases, tenth revision (ICD-10) codes used for the study.**

| **Disease/Procedure** | **ICD-10-CM codes** |
| --- | --- |
| Alcoholic hepatitis | K70.10, K70.11 |
| Obesity Class 1 (BMI 30-34.9) | Z68.30, Z68.31, Z68.32, Z68.33, Z68.34 |
| Obesity Class 2 (BMI 35-39.9) | Z68.35, Z68.36, Z68.37, Z68.38, Z68.39 |
| Obesity Class 3 (BMI ≥ 40) | Z68.41, Z68.42, Z68.43, Z68.44, Z68.45 |
| Upper gastrointestinal bleeding | K20.81, K20.91, K21.01, K22.11, K25.0, K25.2, K25.4, K25.6, K26.2, K26.4, K26.6, K27.0, K27.2, K27.4, K27.6, K28.0, K28.2, K28.4, K28.6, K29.01, K29.21, K29.31, K29.41, K29.51, K29.61, K29.71, K29.81, K29.91, K31.811, K31.82, I85.01, I85.11 |
| Hepatorenal syndrome | K76.7 |
| Septic shock | R65.21 |
| Spontaneous bacterial peritonitis | K652 |
| Hepatic encephalopathy | K72.91, K72.11, K72.01, K76.82 |
| Vasopressor requirement | 3E063XZ, 3E060XZ, 3E053XZ, 3E050XZ, 3E043XZ, 3E040XZ, 3E033XZ, 3E030XZ |
| Intubation/Mechanical ventilation | 5A0945Z, 5A1935Z, 5A1945Z, 5A1955Z, 0BH17EZ, 0BH18EZ |
